# Predicting Quarantine Failure Rates

**DOI:** 10.3201/eid1003.030502

**Published:** 2004-03

**Authors:** Troy Day

**Affiliations:** * Queen’s University, Kingston, Ontario, Canada

**Keywords:** emerging diseases, parasite, contact-tracing, incubation time, epidemiology

## Abstract

Preemptive quarantine through contact-tracing effectively controls emerging infectious diseases. Occasionally this quarantine fails, however, and infected persons are released. The probability of quarantine failure is typically estimated from disease-specific data. Here a simple, exact estimate of the failure rate is derived that does not depend on disease-specific parameters. This estimate is universally applicable to all infectious diseases.

Preemptive quarantine (isolating asymptomatic persons who have had contact with an infected person) is an effective technique for slowing the spread of emerging infectious diseases, but it also results in many uninfected persons being isolated (for examples, see [*1**,**2*]). Health officials must determine an acceptable quarantine duration that balances the social and financial costs of holding potentially uninfected persons for long durations with the risk of releasing an infected person into the general public before he or she displays symptoms, if a shorter duration is used (the quarantine failure rate, 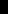). One primary consideration in setting the quarantine duration is the range of observed incubation times. Often the quarantine duration is set to be (approximately) equal to the longest observed incubation period in a sample of *n* infections (*3*). The quarantine failure rate is then monitored through the collection of data on incubation periods throughout the outbreak (*3*).

This approach requires considerable effort, and it must be carried out for each new disease. This assessment of quarantine failure rates is also necessarily retrospective, with the data required for analysis becoming available only after the fact. Here a much simpler approach is derived that requires no data specific to the disease in question. It applies for all possible infectious diseases, and therefore can be employed proactively rather than retrospectively.

If the quarantine duration is chosen to be the longest incubation period in a sample of *n* infections, then the probability, 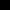 that the quarantine failure rate is no larger than 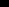 is


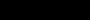
 (equation 1)

for all possible infectious diseases (Appendix). For example, the probability that the quarantine failure rate is no larger than 1% is simply 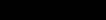. This is valid irrespective of any of the biologic details of the disease of interest. In particular, the form of the underlying probability distribution of incubation times for the disease at hand has no influence on this result.

Often it is of more interest to estimate the quarantine failure rate at a prescribed level of certainty. By rearranging equation 1, we have: with 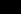 certainty, the quarantine failure rate, 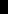, is no larger than 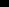, where


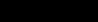
 . (equation 2)

For example, the 95% confidence boundary for the failure rate is simply


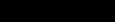
 . Moreover, a point estimate for the failure rate is obtained by calculating the expectation of 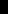:


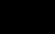
. (equation 3)

Indeed, more generally the probability density of quarantine failure rate, 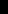, is simply 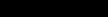 for any infectious disease (Appendix).

The above results also allow one to evaluate the protocol of using the largest incubation period of *n* infected hosts as the quarantine duration. For example, if *n* = 35 (a reasonable value for a newly emerging disease) then the point estimate for 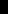 is (equation 3) 1/36 (2.8%), and we have 95% confidence that 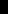 is no larger than 8.2% (equation 2). Thus, a failure rate of 8 in 100 infected persons inadvertently being released from quarantine is within the 95% confidence region. This failure rate is likely unacceptable for highly transmissible diseases.

Alternatively, the above results can be used to determine the sample size, *n*, on which the quarantine duration must be based to ensure that the quarantine failure rate is less than 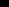 with 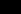 certainty. Rearranging equation 1 yields


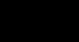
. (equation 4)

This is plotted in the Figure, indicating that enormous sample sizes are required to ensure that the quarantine failure rate is <1%. Together, the above results therefore call for two amendments to preemptive quarantine protocols. First, update the quarantine duration as further infections are observed during an outbreak. This amendment keeps *n* as large as possible. Secondly, set the quarantine duration to be longer than the maximum observed incubation period during the initial stages of the epidemic, when the sample size, *n,* is necessarily small.

**Figure:**
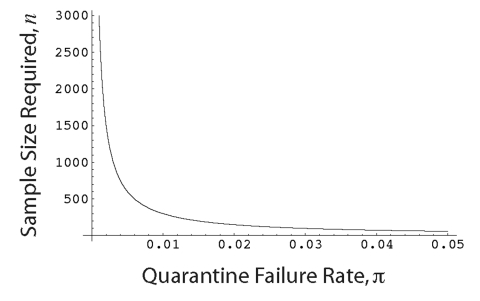
Sample size of infections, *n*, that the quarantine duration must be based on to ensure that the quarantine failure rate is no larger than 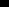 (with 95% certainty). Results assume that the quarantine duration is set equal to the largest incubation period observed in the sample of *n* infections. Curve is plotted using equation 4 with 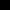
= 0.95.

## Supplementary Material

Appendix Explanation of Formula for Predicting Quarantine Failure Rates
