# Supplementary material for: Predicting Quarantine Failure Rates
Source: Emerg Infect Dis. 2004 Mar;10(3):487–8. doi: 10.3201/eid1003.030502 (PMC3322804; doi:10.3201/eid1003.030502)
Supplement: Appendix — Explanation of Formula for Predicting Quarantine Failure Rates [file 03-0502-app-s1.pdf]

Appendix: Explanation of Formula for Predicting Quarantine Failure Rates

Let L be a random variable denoting the incubation period, and $f(l)$ and $F(l)$ be its probability density function (p.d.f.) and cumulative distribution function. Let M be a random variable denoting the maximum incubation period in a sample of n infections. The p.d.f. of M is then given by $nf(m)F(m)^{n-1}$. In other words, the probability that, after n draws the maximum incubation period is m, is given by the product of the probability that one draw yields an incubation period of exactly m (i.e., $nf(m)dm$) with the probability that the remaining $n-1$ draws all yield incubation periods no larger than m (i.e., $F(m)^{n-1}$). Now introduce a new random variable, $X = F(m)$ (lying in [0,1]), representing the probability that an infected host will have an incubation period no larger than m (where F is the same cumulative distribution function introduced above). The p.d.f. of X is $\frac{d}{dx}\int_0^{G(x)} nf(m)F(m)^{n-1}dm = nx^{n-1}$,

where $G(x)$ is defined to be the inverse

of $F(x)$. The quarantine failure rate is 1-X, and therefore its p.d.f., $p(\phi)$, is

n-n

$p(\phi) = n(1-\phi)^{n-1}$. We then also have $\chi \equiv \int_0^{\pi} n(1-\phi)^{n-1}d\phi = 1-(1-\pi)^n$ and $\bar{\phi} \equiv \int_0^1 \phi n(1-\phi)^{n-1}d\phi = 1/(n+1)$.
